# Influence of body mass index and weight lifting on bicep brachii muscle and distal bicep tendon stiffness evaluated using ultrasound elastography

**DOI:** 10.1186/s12880-020-00531-x

**Published:** 2020-12-10

**Authors:** Mahdi Al-Qahtani, Omar Altuwaijri, Meteb Altaf, Majed Al-Enezi, Mahmoud Abulmeaty, Ravish Javed

**Affiliations:** 1 Biomedical Technology Department, College of Applied Medical Sciences, King Saud University, Riyadh, Kingdom of Saudi Arabia; 2 National Center for Robotics Technology and Intelligent Systems, King Abdulaziz City for Science and Technology, Riyadh, Kingdom of Saudi Arabia; 3 Community Health Sciences Department, College of Applied Medical Sciences, King Saud University, Riyadh, Kingdom of Saudi Arabia

**Keywords:** Ultrasound, Elastography, Strain ratio, Bicep brachii muscle, Distal bicep tendon, Body mass index

## Abstract

**Background:**

This study aimed to investigate the relationship between stiffness of the bicep brachii muscle (BBM) and distal bicep tendon (DBT) and effects of weight lifting (pre- to post-workout changes) among groups with different body mass indexes (BMI).

**Methods:**

Participants were divided into four groups according to BMI: A, underweight (< 18.5 kg/m^2^); B, normal (18.5–24.9 kg/m^2^); C, overweight (25.0–29.9 kg/m^2^); and D, obese (> 30.0 kg/m^2^). All participants were males who were untrained and had sedentary lifestyle without involvement in sports activities for the past 12 months. Ultrasonographic measurements to determine muscle and tendon stiffness was performed on the dominant side (i.e., right side) of the upper extremities in all participants.

**Results:**

Twenty-one healthy and untrained males volunteered to participate in this study; 14 were nonsmokers and 7 were smokers. The mean age and BMI were 22.5 ± 1.5 years and 23.8 ± 6.3 kg/m^2^, respectively. Groups A, B, C, and D had four, ten, four, and three participants, respectively. The BBM thickness did not increase with increase in BMI and was not significantly different (*P* > .05) between groups. The BBM stiffness was significantly different (all *P* < .05) from pre- to post-workout values in all groups, whereas DBT stiffness did not follow the same trend.

**Conclusions:**

Our study revealed that the BBM thickness is independent of BMI. After weight lifting, BBM stiffness in groups A and B increased for BBM compared to those in groups C and D. A similar trend was also recorded for DBT. Weight lifting in concentric and eccentric motions affects the stiffness of the BBM and DBT, thus weight lifting plays a role in adjusting the stiffness of the BBM and DBT.

*Trial registration* The study was approved by ethics committee of the College of Applied Medical Sciences (CAMS 080-3839; March 14, 2018).

## Background

In the upper limbs of the human body, the biceps brachii muscle (BBM) and distal bicep tendon (DBT) play a significant role in simple elbow joint movements, such as lifting and picking up objects and maintaining typing, writing, throwing, and resting positions. The BBM is the longest muscle in the arm, crosses two joints, and is connected to two tendons (long and short) at the proximal side and one tendon at the distal side. Elbow flexion and forearm supination are the most authoritative functions of the BBM and DBT [1], whereas supination of the forearm at the level of the elbow joint is performed solely by the DBT [2]. The BBM can be easily activated with optimum control and full recruitment of the motor unit when subjected to contraction. Pathological conditions, such as shoulder pain, usually involve both the long and short tendons of the BBM [3]. However, the long head of the biceps, due to its anatomical particularities and close functional relationship with the rotator cuff, is more frequently injured than the short head. While injuries of the BBM are rare, overuse and lifting excessive weight can cause muscle fibers to break, leading to severe pain. Injuries of the DBT are also uncommon; only 3% of all bicep tendon injuries (proximal and distal) are caused by partial or complete DBT rupture [4, 5]. Diagnosis of a partial tear of the DBT is complicated, and numerous approaches can be employed, such as ultrasonography (US) [6] and magnetic resonance imaging (MRI) [7]. Complete tears of the DBT are most commonly diagnosed by simply retracting the muscle belly and tendon [8]. Surgical treatment is usually recommended for DBT rupture as the results of conservative treatments are considered unreliable [9].

The BBM is used during upper-limb motion, in which the connected tendons directly and indirectly bear weight and force. Thus, any jerking and sporadic movement can cause damage to both the BBM and DBT. The intactness and strength of the elbow joint, thus the BBM and DBT, are critical to both athletes and non-athletes for optimal elbow function. Individuals who are actively involved in physical or sports activities and those with sedentary lifestyle are at a higher risk of elbow joint-related injuries, due to excessive use in physically active individuals and underuse in individuals with sedentary lifestyle. The stiffness of the BBM and DBT is correlated with body mass index (BMI) and physical activity. The combination of low BMI and sedentary lifestyle is associated with reduced stiffness of the BBM and DBT compared with high BMI and sedentary lifestyle. In contrast, individuals who are physically active with a lower BMI tend to exhibit similar or higher stiffness of the BBM and DBT than those with higher BMI who do not perform any physical activity. The stiffness of the BBM and DBT alone cannot depict the strength or weakness of the muscle independent of BMI, as this is also linked with physical activity; a healthy and lean body is achieved by regular exercise [10]. Muscle strength can be described as the force required in a single effort by a muscle to be used against some form of resistance [11]. Strength training or weight lifting can lead to significant variations in skeletal muscle [12, 13].

Presently, US elastography (USE) is the most appropriate and reasonable approach to measure the stiffness of the BBM and DBT. However, imaging modalities, such as magnetic resonance elastography (MRE) and USE, can be used to identify damage to the BBM and DBT caused by exercise [14, 15] or other physical activities. Both MRE and USE tend to deliver good results. Compared with US, MRE is time-consuming and expensive; however, the traditional US cannot diagnose tissue stiffness. Therefore, USE was developed as an inexpensive, quick, real-time, mobile, and safe alternative to MRI in evaluating tissue stiffness. The method works by identifying the strain induced by applying compression, either manually or automatically, at a particular area in the surrounding tissues.

Previous studies investigating muscle and tendon stiffness provide limited data. To date, there is no study corroborating the relationship of the stiffness of the BBM and DBT and the effects and benefits of weight lifting in individuals with sedentary lifestyle and different BMIs involving external reference material. The present study investigated the association between weight lifting and the stiffness of the BBM and DBT in healthy individuals with sedentary lifestyle. This study aimed to correlate the benefit of weight lifting (15 repetitions each with 2-kg and 5-kg dumbbells) in different groups and its effect on elastographic strain ratios.

## Methods

### Study model

This prospective study was conducted at the Biomedical Technology Department, College of Applied Medical Sciences, King Saud University. The study and its protocols were approved by the ethical review board (number CAMS 080-3839) of the College of Applied Medical Sciences, King Saud University. The guidelines of the Declaration of Helsinki were strictly followed in all experimental settings. Written informed consent was obtained from all participants of the study.

### Participants

The current study recruited male students from the university. Volunteers with any history of corticosteroid treatment, hormone therapy, inflammatory/metabolic diseases, elbow surgery, upper-limb surgery/pain, and weight lifting or, specifically, patients with previous upper-limb injury were excluded from the study. The inclusion criteria were a sedentary lifestyle, lack of involvement in any type of sport or physical activity in the previous year, and Saudi descent. All participants were healthy and active, apart from participating in weight lifting or sports. All participants had right-side dominance.

A mandatory examination session regarding the inclusion criteria was conducted on all volunteers before obtaining actual readings on each volunteer. Thus, 21 participants were finally enrolled and divided into four groups according to BMI: A, underweight (< 18.5 kg/cm^2^); B, normal weight (18.5–24.9 kg/cm^2^); C, overweight (25.0–29.9 kg/cm^2^); and D, obese (> 30.0 kg/cm^2^). The mean age of the volunteers was 22.5 ± 1.5 years to analyze the muscle and tendon in close (or twenties) age range.

### Familiarization to experimental protocols or orientation session for participants

A familiarization session was conducted in front of all potential participants to brief them about the experimental settings and protocols of US and USE. Additionally, one measurement and reading was performed by one of the authors following experimental protocols. Readings of the familiarization session were not included in the analysis. It was clarified that any concerns and questions from the participants related to experimental protocols would be addressed.

### Body mass index and bioelectric impedance analysis

We calculated BMI by measuring the height (in cm) using an immobile stadiometer. Then, this value was entered into body composition analysis equipment (InBody 720, Body Composition Analyser; InBody Corporation Limited, Cerritos, CA, USA), which works on the principle of bioelectrical impedance [16–18]. Besides BMI, skeletal muscle mass (SMM) was also measured using the body composition analyzer.

### Sonographic examinations

**Fig. 1:**
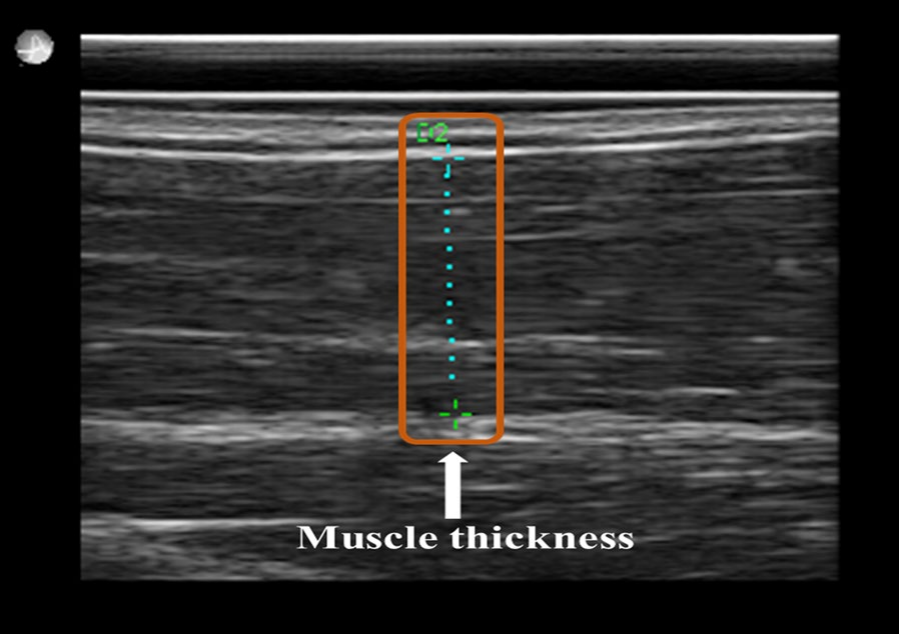
Ultrasonography measurement of bicep brachii muscle thickness at the midpoint

All US examinations were conducted using a high-frequency probe, linear transducer L14-5/38, Ultrasonix SonixTouch Q+ (Analogic Corporation, 8 Centennial Drive, Peabody, MA, USA). Before examination (or measurements) in the current study, all volunteers were subjected to a thorough inspection of the upper limb, starting from the glenohumeral (shoulder) joint until approximately the midpoint of the radius bone for each participant. Moreover, all volunteers were asked to perform all fundamental and possible movements with their arm and forearm to identify if any abnormality in the proximal and distal tendons was present. Participants were seated in an adjustable-height chair next to a table. The dominant arm was extended 180º and placed on the table with a pillow underneath for comfort. Thickness was measured at the midpoint of the biceps brachii (Fig. 1), with the US probe notch facing the glenohumeral joint over the skin without exerting any pressure. Earlier, volunteers were asked to perform maximum elbow flexion, and the midpoint was marked at the center of the bicep brachii belly. All parameters of the US machine were kept constant, even the pillow used for the study was the same in all volunteers, as reproducibility is operator dependent and readings can change with slight variations. Thus, readings were obtained one after another without any break. To ascertain that a fixed pressure is applied on the skin and the same amount of strain is produced each time all measurements were obtained at four-bar indication, which can be seen on the bottom left of Fig. 4, it is the strain level indicator provided by the manufacturer of the US machine.

### Strain elastography measurements

**Fig. 2:**
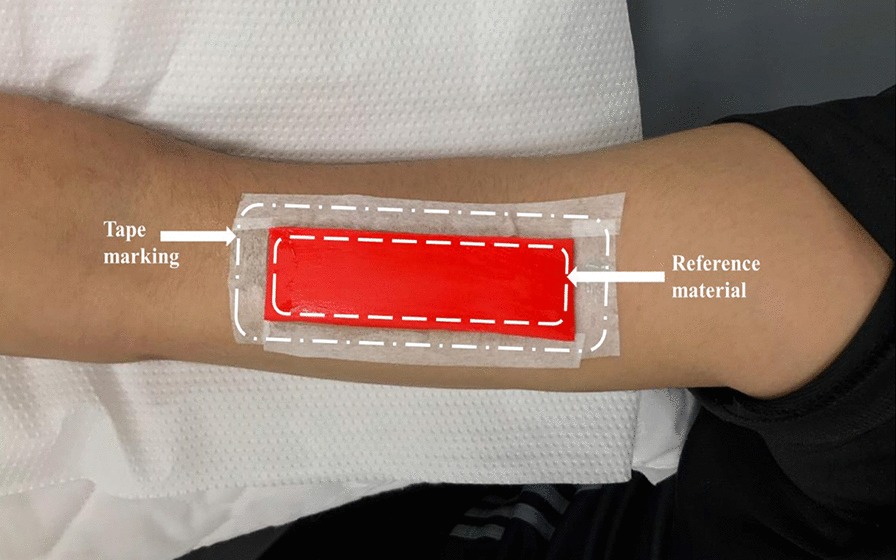
Reference material and tape used to mark the bicep brachii muscle

**Fig. 3:**
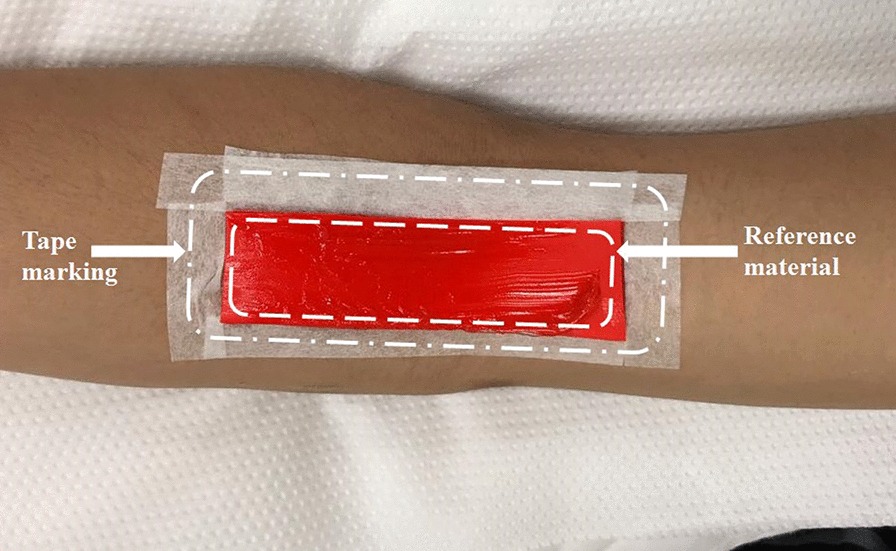
Reference material and tape used to mark the distal bicep tendon

**Fig. 4:**
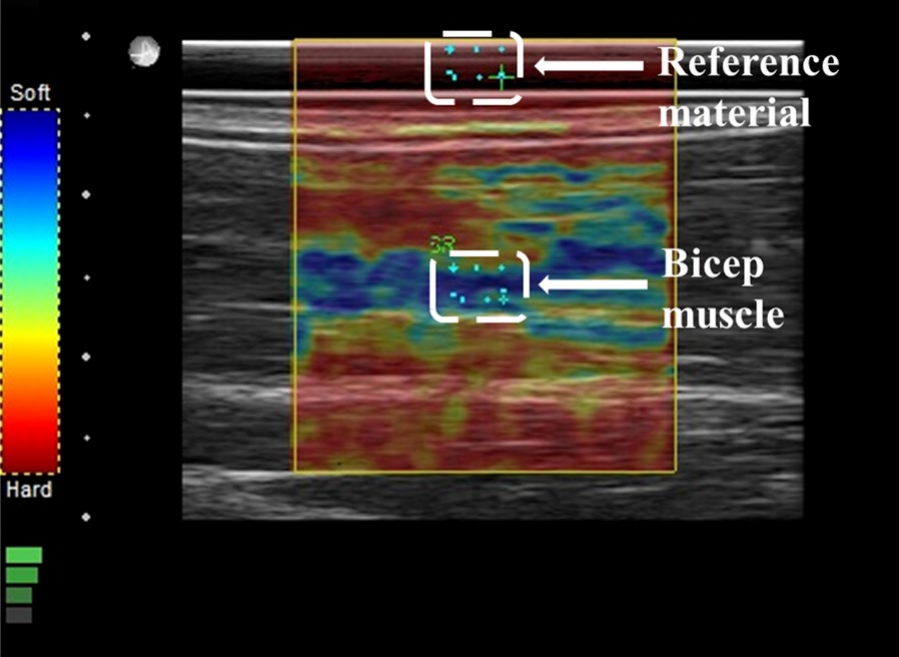
Ultrasonography measurement of bicep brachii muscle strain ratio using the reference material

Strain elastography (SE) measurements for the BBM and DBT were obtained using the same instrument (selecting Elasto Mode) as previously used for US measurements. All SE readings of the BBM were recorded by placing the transducer over the external reference material (with dimensions 79 × 24.5 × 2 mm) on the belly of the BBM. The elasticity of the external reference material was 4.01 ± 1.13 MPa. Marks were made around the borders of the reference material with the use of a tape (Fig. 2). The reference material is commercially available. The use of external reference material is considered a more valid and reliable approach, especially when the number of participants is more than one, to overcome the limitation of subcutaneous fat tissue, which changes in every participant. The external reference material had a constant elasticity, in contrast to the BBM. The US transducer was placed in the longitudinal plane over the bicep brachii belly, with the notch of the US probe facing the shoulder of the examinee. All SE readings were measured at the center of the probe placement. A similar protocol was followed for the measurement of the DBT by SE (Fig. 3). To minimize the chance of error and avoid under- or overestimation of stiffness for all volunteers, the same external reference material was used and strain values were measured at four-bar indication, which can be seen on the bottom left of Fig. 4, which is the strain level indicator provided by the manufacturer of the US machine. However, there can still be a chance of little or no variation when measuring the strain ratios due to the amount of subcutaneous tissue that can differ for each individual.

### Bicep brachii muscle measurement arrangement

We conducted SE readings in three phases of the BBM. In the initial phase, three readings were obtained at the BBM for each participant before lifting any weight (Fig. 4). In the second phase, after rest, participants were asked to lift a 2-kg dumbbell 15 times in a relaxed seated position. While seated, each participant was instructed to start from an elbow extension angle of approximately 0° to an elbow flexion of approximately 90°. Fifteen repetitions were performed in this manner, and three SE readings of the BBM were obtained in the same position. In the final phase, after a brief rest, a 5-kg dumbbell was lifted 15 times as previously described, and three SE readings were obtained for the BBM. Dumbbell weights of 2 and 5 kg were used in all groups as this weight range is usually encountered in daily life, regardless of whether an individual is involved in weight lifting or has a sedentary lifestyle, and thus can easily be lifted by all participants. Fixed dumbbell weights were used in all groups to ensure the same amount of weight-lifting impact on the BBM. The mean of the three readings was calculated for each phase to increase intraobserver reliability and decrease deviation and risks of error.

### Distal bicep tendon measurement arrangement

**Fig. 5:**
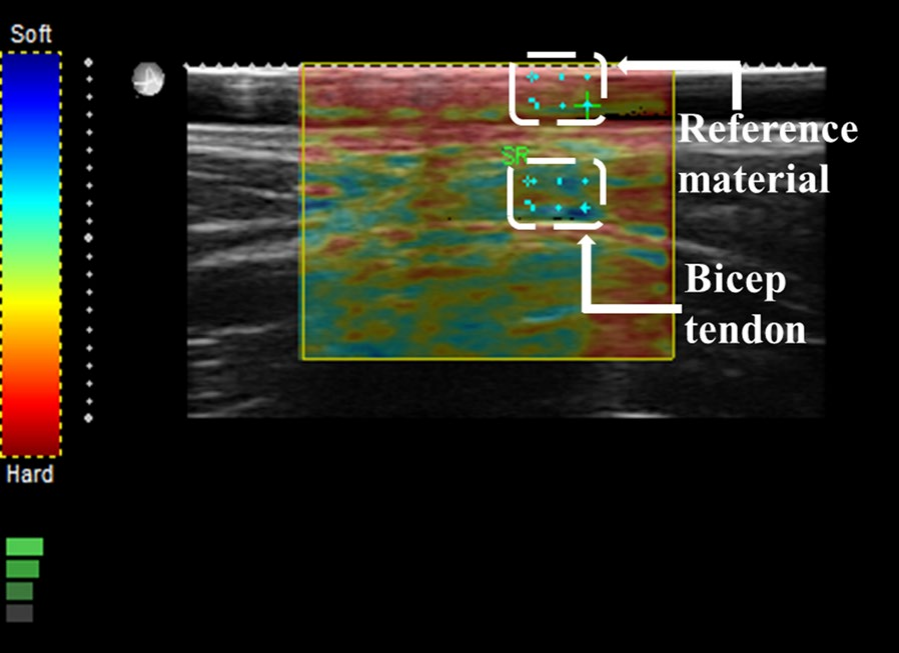
Ultrasonography measurement of distal bicep tendon muscle strain ratio using the reference material

To measure the DBT by SE, each participant underwent three phases (as for the BBM). The DBT readings were measured by asking the participant to move their forearm in maximum supination for better visualization. This protocol was followed in all volunteers for DBT measurements. The SE readings for all three phases were calculated for each participant just after taking readings of the BBM, with similar protocol and settings (Fig. 5).

### Inclusion of external reference material

An external reference was used to maintain consistency and set a common reference for all SE values (for both the BBM and DBT). A silicon rubber was purchased from a commercial source. The dimensions and elasticity of the reference material were 80 × 24 × 2 and 4.01 ± 1.13 MPa, respectively. Two regions of interest were selected in calculating the value of SE in the case of BBM (BBM/adjacent external reference material; Fig. 4). Similarly, Fig. 5 shows the two regions of interest selected for calculating the SE value for the DBT (DBT/adjacent external reference material).

### Statistical analysis

We used SPSS Statistics version 25 software for Windows (IBM Corporation, Armonk, NY, USA) in the statistical analysis. One-way analysis of variance was performed using Tukey’s honestly significant difference test to evaluate the level of significance among the three phases for each group. Differences between the groups were found using *t* test, while post hoc Tukey’s test was performed to determine the level of significance between them. All values were presented as mean ± standard deviation with a *P* value of 0.05 indicating significant difference.

## Results

We enrolled 21 participants aged 22.5 ± 1.5 years (range 21–28 years). The mean weight, height, and BMI of all 21 participants were 70.1 ± 18 kg, 171.8 ± 6.0 cm, and 23.8 ± 6.3 kg/cm^2^, respectively. The age ranges for groups A (n = 4), B (n = 10), C (n = 4), and D (n = 3) were 22–23, 21–28, 21–24, and 22–22 years, respectively; seven participants were smokers and 14 were nonsmokers. The mean BMIs for each group was 17.4 ± 0.7, 21.6 ± 2.1, 27.2 ± 0.8, and 35.4 ± 6.3 kg/cm^2^ for groups A, B, C, and D, respectively. The SE values for the BBM and DBT before and after weight lifting showed that strain values significantly differed according to BMI. A statistically significant difference was observed for most factors between the four groups.

**Fig. 6:**
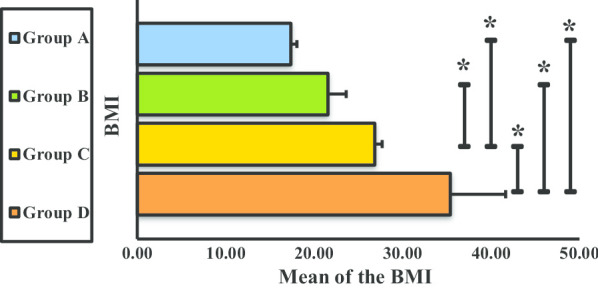
Body mass index of groups A, B, C, and D. *P < 0.05. *BMI* body mass index, *Group A* underweight (< 18.5 kg/cm^2^), *Group B* normal weight (18.5–24.9 kg/cm^2^), *Group C* overweight (25.0–29.9 kg/cm^2^), *Group D* obese (> 30.0 kg/cm^2^)

The mean BMI significantly differed between all groups, except for groups A and B (Fig. 6).

**Fig. 7:**
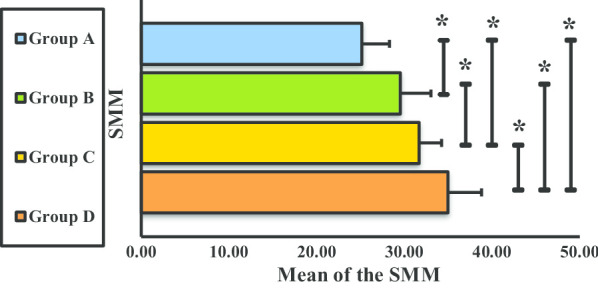
Skeletal muscle mass of groups A, B, C, and D. *P < 0.05. *SMM* skeletal muscle mass, *Group A* underweight (< 18.5 kg/cm^2^), *Group B* normal weight (18.5–24.9 kg/cm^2^), *Group C* overweight (25.0–29.9 kg/cm^2^), *Group D* obese (> 30.0 kg/cm^2^)

The mean SMM measurements significantly differed between groups (Fig. 7).

**Fig. 8:**
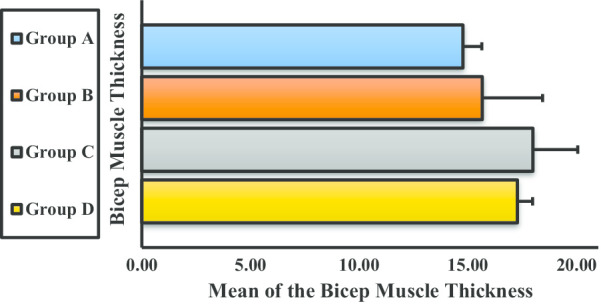
Mean thickness of the biceps brachii muscle for groups A, B, C, and D. Definitions: Group A, underweight (< 18.5 kg/cm^2^); Group B, normal weight (18.5–24.9 kg/cm^2^); Group C, overweight (25.0–29.9 kg/cm^2^); Group D, obese (> 30.0 kg/cm^2^)

Figure 8 shows the mean BBM thickness for each group before weight lifting. Although the thickness increased from groups A to B and B to C, the mean thickness of group D was found to be decreased compared with that of group C. None of the differences were statistically significant.

**Fig. 9:**
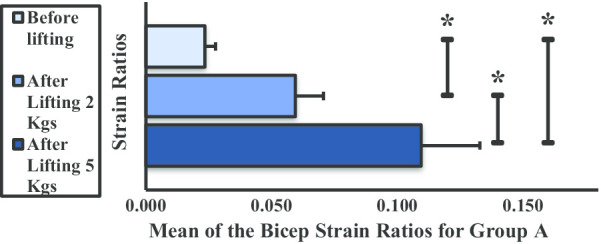
Mean strain ratios of the biceps brachii muscle for group A. *P < 0.05

The mean strain ratio of the BBM for group A showed an increasing trend after lifting of 2- and 5-kg dumbbells (Fig. 9). Additionally, a significant difference was noted between each phase.

**Fig. 10:**
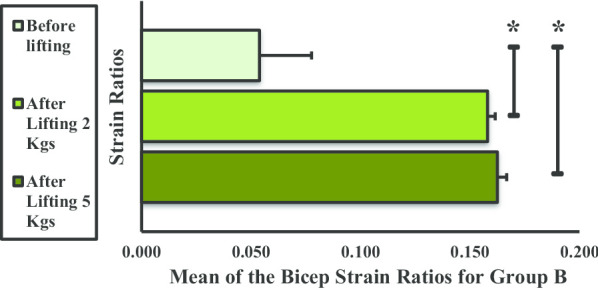
Mean strain ratios of the biceps brachii muscle for group B. *P < 0.05

The mean strain ratio of the BBM for group B also showed an increasing trend after lifting the 2- and 5-kg dumbbells (Fig. 10). Furthermore, a significant difference was observed before and after lifting the 2-kg weight and before and after lifting the 5-kg weight. No significant difference was observed between lifting the 2-kg and 5-kg weight.

**Fig. 11:**
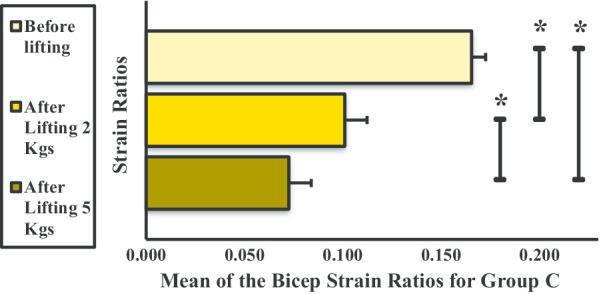
Mean strain ratios of the biceps brachii muscle for group C. *P < 0.05

In group C, the strain ratio of the BBM decreased when subjected to weight lifting. The strain ratios in each phase were significantly different before and after lifting 2 kg, before and after lifting 5 kg, and between lifting 2 kg and 5 kg weight (Fig. 11).

**Fig. 12:**
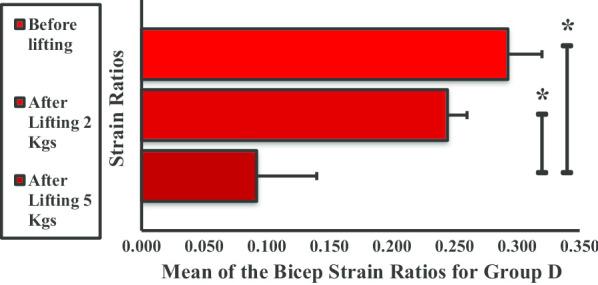
Mean strain ratios of the biceps brachii muscle for group D. *P < 0.05

The mean strain ratio for the BBM in group D dramatically decreased when subjected to weight lifting (Fig. 12). The strain ratios were significantly different before and after lifting 2 kg and before and after lifting 5 kg. No significant difference was observed between lifting 2 kg and 5 kg.

**Fig. 13:**
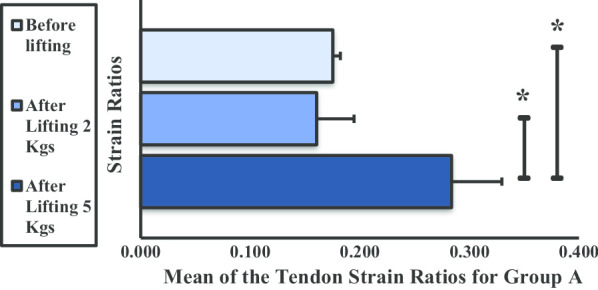
Mean strain ratios of the distal bicep tendon for group A. *P < 0.05

Figure 13 shows the mean strain ratio of the DBT for group A. The values showed variation when subjected to lifting 2 and 5 kg dumbbells. A significant difference was observed from before lifting to after lifting 5 kg and after lifting 2 kg to lifting 5 kg. However, no significant difference was observed before lifting and after lifting 2 kg dumbbells.

**Fig. 14:**
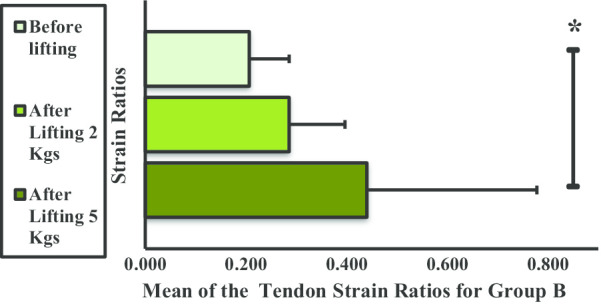
Mean strain ratios of the distal bicep tendon for group B. *P < 0.05

In group B, the mean strain ratios for the DBT increased when subjected to weight lifting, although these differences were not significant (Fig. 14). A significant difference was noted between the strain ratios before lifting and after lifting 5 kg.

**Fig. 15:**
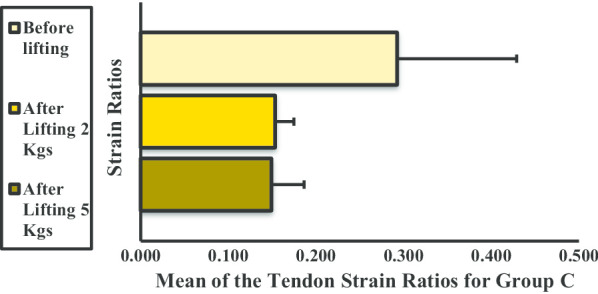
Mean strain ratios of the distal bicep tendon for group C

As Fig. 15 shows, the mean strain ratios for the DBT in group C showed no significant difference in any phase, although the value decreased across the three phases.

**Fig. 16:**
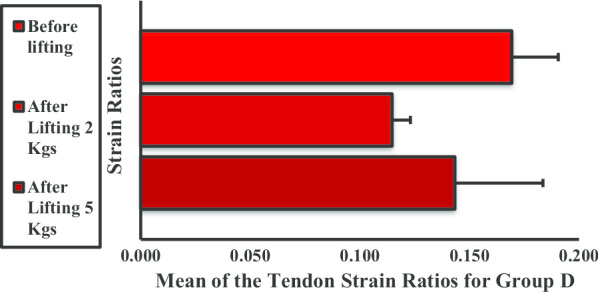
Mean strain ratios of the distal bicep tendon for group D

The mean strain ratios of the DBT for group D showed no significant difference when subjected to weight lifting (Fig. 16) but exhibited irregular changes when subjected to lifting 2 or 5 kg dumbbells compared with that before lifting.

A significant difference was noted among groups A, B, C, and D for mean values of strain ratios in BBM before weight lifting but not after lifting 2 and 5 kg. In the case of DBT, the variation in mean values of strain ratios was only statistically significant in groups B and D after lifting 2 kg (Table 1).

**Table 1:** Mean strain ratios of bicep brachii muscle and distal bicep tendon in all groups

	Groups				
	A (Underweight)	B (Normal)	C (Overweight)	D (Obese)	
Before lifting weight	0.024^*,†^	0.054^§,#^	0.166^*,§,¶^	0.293^†,#,¶^	Bicep brachii muscle
After lifting 2 kg	0.060	0.158	0.101	0.244	
After lifting 5 kg	0.110	0.163	0.073	0.092	
Before lifting weight	0.175	0.207	0.293	0.169	Distal bicep tendon
After lifting 2 kg	0.161	0.285^Δ^	0.153	0.115^Δ^	
After lifting 5 kg	0.284	0.440	0.149	0.143	

Key: Symbols *, †, §, #, ¶, Δ indicate the presence of significant difference (*P* < 0.05) between groups

## Discussion

The aim of this study was to scrutinize the association of the effects of weight lifting on the BBM and DBT concerning BMI. The current study results revealed that SE measurements of BBM showed significant variation when exposed to weight lifting, while SE measurements of DBT showed significant variation in only a few scenarios. Variations in the mean SE values seemed to be correlated with the mean BMI of various groups, all of them having a sedentary lifestyle. To date, there are no data on the effect of weight lifting (in concentric and eccentric motions, using two specific weights) on the SE (evaluated using an external reference) of the BBM and DBT.

In groups A and B, there was a significant increase in BBM mean strain ratio (or stiffness) before and after lifting 2- and 5-kg dumbbells. Newham et al. [19] reported that, in the first 3 days of exercises, eccentric movements can cause instant ultrastructural damage to the sarcomeres of the muscle fibers, affecting myofibrillar structures. The intramuscular water content level increases following repetitive muscle contractions [20, 21]. Thus, the increase in BBM stiffness is due to a combination of factors, including change in the osmotic grade of the vascular and extravascular spaces, production of metabolites in the affected muscle, and induced edema coupled to increased blood flow (intramuscular) due to increased permeability and capillary pressure [22–24]. Weight lifting causes microfiber injury in the BBM that led to an increase in stiffness in groups A and B. This results in increased muscular pressure due to fluid accumulation. Microscopic muscle damage is a consequence of increased stress in the muscle fiber, which produces microtears as the muscle is stretched by intense concentric and eccentric muscle movements [25]. Few studies have confirmed that concentric and eccentric muscle contractions cause increased muscle stiffness [26, 27]. It has been observed that weight lifting is strenuous to unaccustomed exercises or movements that affect the stiffness and mechanical properties of muscles and the joint activity and the movements of an individual for 24–72 h. In a study by Agten et al. [28], sarcolemmal breakup and opening of mechanosensitive cell membrane channels triggered by eccentric exercise cause accumulation of intracellular Ca^2+^ and Na^2+^ ions. This causes increased cellular damage, inflammation, and edema and transient reduction in functionality; however, these ion influxes are also responsible for stimulus-induced muscle hypertrophy [29]. To remove necrotic cells, inflammatory cells (primarily neutrophils) penetrate skeletal muscles in concentric and eccentric exercises of 45 min to 2 h in duration [30, 31]. In the present study, the increased stiffness observed in groups A and B might be due to the increase in blood flow and extracellular muscle edema following weight lifting. The increase in stiffness of BBM after weight lifting was approximately equal in group A from before lifting to after lifting 2 kg and from after lifting 2 kg to after lifting 5 kg. The significant difference in BBM stiffness across all three phases could be explained by the absence of sufficient SMM to counter the concentric and eccentric exercise movement. Sufficient skeletal muscle comprises 40% of human body weight [32] and was found to be not present in group A volunteers by the body composition analyzer. In group B, a significant difference was observed in BBM stiffness from before lifting to after lifting 2 kg, the difference from after lifting 2 kg to after lifting 5 kg was not significant in group B, and group B has higher SMM than group A, as it was measured using the body composition analyzer. The results of a previous study by Tomlinson et al. relating to increased adiposity and muscle stiffness among overweight individuals compared with normal or underweight individuals corroborate our findings [33]. Groups C and D have higher BMI; thus, the presence of higher fat infiltration [34] and higher SMM might be the cause of decrease in stiffness in these two groups. Moreover, in the earlier study, weight lifting causes decreased SMM [35]. The hypothesis is that the greater the BMI, the lower the BBM stiffness. This may be attributed to obesity-related changes in skeletal muscles. In group C, the presence of higher SMM and a decrease in stiffness values with activity were recorded. In group D, no significant difference was observed in BBM stiffness before lifting and after lifting 2 kg because of the presence of higher SMM, which was also higher than group C.

The current study reveals that DBT stiffness varies depending on the BMI. A previous study by Bohm et al. [36] reported that tendons are responsive to loading procedures, which corroborates our findings by showing different stiffness values for DBT readings. Another study by Kubo et al. [37] reported stiffness of the tendon to be proportional to the time spent exercising; thus, after lifting 2 and 5 kg weights, there was a difference in DBT stiffness among all groups. A study by Siu et al. [38] revealed that stiffness of the tendon is increased by exercise, which validates our findings that groups A and B exhibited significant differences in DBT stiffness following exercise. However, this result was not observed in groups C and D, indicating that tendon composition is different than in groups A and B due to BMI; a previous tendon study validates our results [39]. In the present study, stiffness decreased with the increased weight used for concentric and eccentric weight lifting. Groups C and D represent overweight and obese participants, respectively; thus, their tendon has more stiffness and less likely to be affected by 2- and 5-kg dumbbell weight lifting. A possible reason for the decreased stiffness in overweight and obese individuals is the changes in the structure of the tendon that impairs the movement of interstitial fluid in response to weight lifting [40]. The lack of significant differences in BBM and DBT stiffness in groups C and D is caused by the difference in the SMM. Despite the increase in the SMM, obesity is associated with an increased amount of type II muscle fiber, reduction in satellite cell activation, and insulin sensitivity, which impairs muscular regeneration. Moreover, obesity-associated low-grade chronic inflammation could impair skeletal muscle protein synthesis [41].

Our study involved Saudi men in their twenties with sedentary lifestyle. As muscles and tendons [42] develop and grow differently in females than males, future studies on muscles and tendon stiffness in females would be informative. To further strengthen the relationship between BBM and DBT stiffness with BMI, new studies involving children, elderly participants, athletes, and unhealthy individuals are required, as they were excluded in the present study. Furthermore, the limited reproducibility is a weakness of USE as it is operator dependent. Moreover, an increased or equal sample size for each group is highly recommended for future studies, as the present study has a limited number of volunteers in one group. Another study can be conducted to relate the stiffness with the subcutaneous soft tissue to overcome the limitations of the BMI measurements. However, the same amount of subcutaneous soft tissue present in two different individuals can lead to similar or different stiffness readings.

## Conclusions

Our study provides insight into the stiffness of muscles and tendons following weight lifting and is the first to analyze this parameter of the BBM and DBT after weight lifting concerning BMI using SE. Generally, weight lifting in concentric and eccentric motions affects the stiffness of the BBM and DBT, and the direction of this effect depends on the BMI, with stiffness increasing in individuals with lower BMI and decreasing in individuals with higher BMI. In this study, we attempted to establish an association between the BMI and muscle stiffness and track the changes in the SMM by bioelectric impedance analyzer during weight lifting. Weight lifting plays a role in adjusting (increasing or decreasing) the BBM and DBT stiffness.

## Data Availability

The datasets generated and/or analyzed in the current study are not publicly available due to patient privacy protection but are available from the corresponding author on reasonable request.

## Abbreviations

BBM: Bicep brachii muscle
DBT: Distal bicep tendon
BMI: Body mass index
US: Ultrasound
MRI: Magnetic resonance imaging
MRE: Magnetic resonance elastography
SMM: Skeletal muscle mass
USE: Ultrasound elastography
SE: Strain elastography
